# Genome-wide association study of HLA-DQB1*06:02 negative essential hypersomnia

**DOI:** 10.7717/peerj.66

**Published:** 2013-04-16

**Authors:** Seik-Soon Khor, Taku Miyagawa, Hiromi Toyoda, Maria Yamasaki, Yoshiya Kawamura, Hisashi Tanii, Yuji Okazaki, Tsukasa Sasaki, Ling Lin, Juliette Faraco, Tom Rico, Yutaka Honda, Makoto Honda, Emmanuel Mignot, Katsushi Tokunaga

**Affiliations:** 1 Department of Human Genetics, Graduate School of Medicine, The University of Tokyo, Tokyo, Japan; 2 Yokohama Clinic, Warakukai Medical Corporation, Yokohama, Japan; 3 Department of Psychiatry, Mie University School of Medicine, Mie, Japan; 4 Metropolitan Matsuzawa Hospital, Tokyo, Japan; 5 Graduate School of Education, The University of Tokyo, Tokyo, Japan; 6 Stanford Center for Sleep Sciences and Medicine, Stanford University School of Medicine, Palo Alto, CA, USA; 7 Department of Somnology, Tokyo Medical University, Tokyo, Japan; 8 Sleep Research Project, Department of Psychiatry and Behavioral Sciences, Tokyo Metropolitan Institute of Medical Science, Tokyo, Japan

**Keywords:** *SPRED1*, Essential hypersomnia, *NAP5*, *NCKAP5*, *CRAT*, HLA-DQB1*06:02, Narcolepsy with cataplexy, Narcolepsy, EHS

## Abstract

Essential hypersomnia (EHS), a sleep disorder characterized by excessive daytime sleepiness, can be divided into two broad classes based on the presence or absence of the HLA-DQB1*06:02 allele. HLA-DQB1*06:02-positive EHS and narcolepsy with cataplexy are associated with the same susceptibility genes. In contrast, there are fewer studies of HLA-DQB1*06:02 negative EHS which, we hypothesized, involves a different pathophysiological pathway than does narcolepsy with cataplexy. In order to identify susceptibility genes associated with HLA-DQB1*06:02 negative EHS, we conducted a genome-wide association study (GWAS) of 125 unrelated Japanese EHS patients lacking the HLA-DQB1*06:02 allele and 562 Japanese healthy controls. A comparative study was also performed on 268 HLA-DQB1*06:02 negative Caucasian hypersomnia patients and 1761 HLA-DQB1*06:02 negative Caucasian healthy controls. We identified three SNPs that each represented a unique locus— rs16826005 (*P* = 1.02E-07; *NCKAP5*), rs11854769 (*P* = 6.69E-07; *SPRED1*), and rs10988217 (*P* = 3.43E-06; *CRAT*) that were associated with an increased risk of EHS in this Japanese population. Interestingly, rs10988217 showed a similar tendency in its association with both HLA-DQB1*06:02 negative EHS and narcolepsy with cataplexy in both Japanese and Caucasian populations. This is the first GWAS of HLA-DQB1*06:02 negative EHS, and the identification of these three new susceptibility loci should provide additional insights to the pathophysiological pathway of this condition.

## Introduction

Hypersomnias are one of the important classes of sleep disorders, patients are manifested by recurring episodes of excessive daytime sleepiness (EDS) that are not due to tiredness. Hypersomnia can be a symptom of other sleep disorders such as narcolepsy with cataplexy, essential hypersomnia (EHS), sleep apnea and idiopathic hypersomnia.

In 1986, Yutaka Honda described a group of patients with a narcolepsy-like condition that lacked cataplexy; he designated this condition essential hypersomnia syndrome (EHS) (Honda et al., 1986). EHS is characterized by excessive daytime sleepiness features that are indistinguishable from those of narcolepsy such as shorter episodes of irresistible daytime sleepiness, feelings of refreshment after short naps, and the absence of prolonged nocturnal sleep time. Patients with EHS show frequent sleep-onset rapid eye movement (REM) periods (SOREMPs) when a multiple sleep latency test (MSLT) is performed (2001). Honda and his colleagues later reported that the symptomatic characteristics of these patients are different from those of patients with classical idiopathic hypersomnia syndrome (IHS); reportedly, IHS sleep patterns include long naps and prolonged nocturnal sleep (Roth, 1976). Based on the criteria in the International Classification of Sleep Disorders second edition (ICSD-2) (American Academy of Sleep Medicine, 2005), the diagnostic criteria for EHS correspond to those for narcolepsy without cataplexy and most of those for IHS without long sleep. Previously we showed that approximately 40% of the patients with EHS carry the HLA-DQB1*06:02 allele but that 12% of the general Japanese population and 100% of the patients with narcolepsy with cataplexy carry this allele (Mukai et al., 2003; Miyagawa et al., 2009). Additionally several reports from other groups also indicate that HLA-DQB1*06:02 is associated with the pathogenesis of narcolepsy with cataplexy (Mignot et al., 2001; Juji et al., 1984; Marcadet et al., 1985); given these finding, we believe that the pathogenesis of EHS may partially differ from that of narcolepsy with cataplexy. In addition, study has also suggested that EHS is likely to be a member of hypersomnia based on the differences in genetic composition of EHS and narcolepsy with cataplexy, milder disease severity of EHS, and the superior treatment response of EHS (Mukai et al., 2003). Based on Epworth Sleepiness Scale (ESS) and MSLT evaluations, the severity of EDS is significantly milder in EHS than in narcolepsy with cataplexy (Komada et al., 2005).

Several studies carried out by our group demonstrate that common susceptibility genes exist between HLA-DQB1*06:02 positive EHS and narcolepsy with cataplexy patients, suggesting a common etiological pathway might exist for HLA-DQB1*06:02 positive EHS and narcolepsy with cataplexy patients. For example, *TCRA* is reported to be strongly associated with narcolepsy with cataplexy (Hallmayer et al., 2009) and our replication study indicated that *TCRA* is also associated with HLA-DQB1*06:02 positive EHS (Miyagawa et al., 2010). In contrast, *CPT1B* and *CHKB*, which are reportedly associated with narcolepsy with cataplexy in a Japanese population (Miyagawa et al., 2008), are also reportedly associated with both HLA-DQB1*06:02-positive EHS and EHS in patients lacking HLA-DQB1*06:02 (designated here as HLA-DQB1*06:02-negative EHS) (Miyagawa et al., 2009). Taken together these findings indicate that *CPT1B* and *CHKB* have a broader functional role in hypersomnias as general.

Based on the hypothesis that HLA-DQB1*06:02 negative EHS has a different etiological pathway from that of narcolepsy with cataplexy, we aimed to identify commonly occurring genetic variants that are associated with susceptibility to HLA-DQB1*06:02 negative EHS in a Japanese study population. To our knowledge, there is no published report of a genome-wide association study (GWAS) on any hypersomnia in any human population other than those GWASs on narcolepsy with cataplexy.

## Materials and Methods

### Subjects

EHS was diagnosed based on the following three clinical items in central nervous system hypersomnias: (i) recurrent daytime sleep episodes that occur basically everyday over a period of at least 6 months; (ii) absence of cataplexy; (iii) the hypersomnia is not better explained by another sleep disorder, medical or neurological disorder, mental disorder, medication use or substance use disorder (Mukai et al., 2003; Komada et al., 2005; Miyagawa et al., 2009; Honda et al., 1986). We focused our studies on patient with EHS, but lacking HLA-DQB1*06:02, because previous studies (Miyagawa et al., 2010; Honda et al., 1986; Mukai et al., 2003) indicated that HLA-DQB1*06:02 negative EHS is essentially different from HLA-positive EHS and narcolepsy with cataplexy. In this GWAS, we recruited 125 individuals who were given a diagnosis of EHS at a clinic affiliated with Neuropsychiatric Research Institute of Japan and 562 Japanese individuals as healthy controls. All genomic DNA samples were genotyped using Affymetrix Genome-Wide SNP Array 6.0 platform. In order to validate the accuracy of the top SNPs from the Affymetrix Genome-Wide SNP Array 6.0 platform, three SNPs (rs11854769, rs12471007 and rs10988217) were genotyped by TaqMan assay.

In order to elucidate the effects of susceptibility SNPs found in Japanese EHS GWAS samples in other population, a collaboration study with the Center for Narcolepsy, Stanford University School of Medicine, was carried out. The comparative study of a Caucasian population focused on patients with 268 HLA-DQB1*06:02-negative hypersomnias and 1761 HLA-DQB1*06:02-negative healthy controls. All subjects had given written informed consent for their participation in these studies in accordance with the process approved by ethics committees of the University of Tokyo and Stanford University.

### HLA genotyping

*HLA-DQB1* genotyping for EHS samples were performed using Luminex Multi-Analyte Profiling System (xMAP) together with the WAKFlow HLA typing kit (Wakunaga, Hiroshima, Japan). Briefly, target DNA was amplified by PCR (polymerase chain reaction) with biotinylated primers. The PCR amplicon was then denatured and hybridized to complementary oligonucleotide probes immobilized on fluorescent coded microsphere beads. In the meantime, biotinylated PCR products were labeled with phycoerythrin-conjugated streptavidin, and finally HLA typing was examined by Luminex 100 (Luminex, Austin, TX).

### Genotyping and quality control

Genotype calling was performed using Affymetrix Genotyping Console 4.0, which employs the Birdseed genotype calling algorithm for Affy 6.0 (*n* = 687). Samples with a low quality control call rate (typically <95%) were excluded before performing the full clustering analysis of genotypes. For each Birdseed genotype call, SNPs with call rates <99%, SNP that shown deviation from Hardy-Weinberg (*P* < 0.001) in controls, monomorphic SNP, and sex-chromosome SNPs were excluded from subsequent analysis. Cluster plots of the top 100 SNPs that showed the strongest association were checked visually and ambiguously clustered SNPs were excluded; only one SNP was excluded based on this cluster criterion. In the absent of independent Japanese EHS samples for replication, we validated the accuracy of randomly selected SNPs genotyped by Affymetrix 6.0 platform using a TaqMan assay.

### Statistical analysis

Association analysis of SNPs were analyzed using an allelic model, a dominant model, a recessive model, or a Cochran–Armitage trend test using PLINK (v1.07) (Purcell et al., 2007). Population stratification within the Japanese patients with EHS was evaluated based on the genomic inflation factor (λ), which was calculated from the median of the Cochran–Armitage trend test. The quantile-quantile (Q–Q) plot was plotted with expected distribution of association test statistics under null distribution across the Cochran–Armitage trend observed *P*-values with *R* statistical environment version 2.9.0. Peta odd ratio was used to calculate odd ratio for any contingency column with counting of 0, online calculator is available at http://www.hutchon.net/ConfidORnulhypo.htm. Manhattan-plot was generated using Haploview (v4.1) (Barrett et al., 2005). For the Caucasian comparative study, an allelic, a dominant, and a recessive model were each assessed using a 2-tailed chi-square test. The eQTL analyses were performed based on data from the Sanger Institute GENEVAR project (Yang et al., 2010); these data are based on three cell types (fibroblast, lymphoblastoid cell line and T-cell) of 75 unrelated Western European origin individuals (Dimas et al., 2009). The SNPExpress Database (Heinzen et al., 2008), which is based on 93 autopsy-collected cortical brain tissue with no defined neuropsychiatric condition and 80 peripheral blood mononucleated cell (PMBC) samples collected from healthy donors, was also used as a reference for eQTL analyses. Relationships between the genotypes of candidate loci and the expression levels of nearby genes and transcripts were also examined.

### Imputation

MACH version 1.0 (Li et al., 2010) was used to estimate haplotypes, map crossover and error rates using 50 iterations of the Markov chain Monte Carlo algorithm. By employing genotype information from HapMap Phase II (release 23) database (International Hapmap Consortium, 2005), maximum likelihood genotypes were generated. For the quality control of the imputed genotypes, imputed genotypes with the estimated *r*2 > 0.3 were retained. Imputed genotypes were re-analyzed by allelic, dominant, and recessive models and the Cochran–Armitage trend test utilizing PLINK 1.7. Regional association plots were generated using LocusZoom (Pruim et al., 2010).

## Results

This is the first reported GWAS that aimed to identify common genetic variants associated with HLA-DQB1*06:02 negative EHS. Specifically, we sought to identify susceptibility loci, other than HLA loci, for EHS using a collection of samples that lacking the HLA- DQB1*06:02. For this GWAS, we recruited 125 individuals who were given a diagnosis of EHS at a clinic that is affiliated with the Neuropsychiatric Research Institute of Japan and 562 Japanese healthy controls. Genomic DNA samples from each individual were genotyped using the Affymetrix Genome-Wide SNP Array 6.0 platform. After quality controls (see Methods for details), statistical tests were performed on 508,366 remaining SNPs. Population stratification was accessed by calculating the genomic inflation factor (λ); the λ of this data set was 1.008; this finding indicated that errors resulting from population stratification, cryptic relatedness, or both were unlikely (Fig. S1). A genome-wide Manhattan plot was drawn using the chromosomal positions of individual SNPs (*x*-axis) and the negative logarithm of *P* values calculated with the Cochran–Armitage trend test (*y*-axis) (Fig. S2).

We identified one genomic region, 2q21.2, that contained clustered SNPs that were significantly associated (*P*-value < 5 × 10^−7^) with increased risk of EHS in this Japanese population (Fig. S2 and Table 1). Additionally, we chose to further investigate 9q34 because of its functional importance.

The SNP rs16826005 had the lowest *P*-value (1.02E-07, per-allele odds ratio (OR) of 1.89 with 95% confidence interval (CI) of 1.43–2.50) of all SNPs assessed; rs16826005 was located within a 19-kb linkage disequilibrium (LD) block on chromosome 2q21.2 (Table 1). This LD-block covered the intronic region of *NCKAP5* (NCK-associated protein 5) gene. Imputation analysis of this region revealed modest associations between HLA-DQB1*06:02 negative EHS and SNPs in the *NCKAP5* gene. This finding indicated that *NCKAP5* may play a causative role in EHS pathogenesis. Expression data for *NCKAP5* is not readily available in the Gene Variation Database (GeneVar) (Yang et al., 2010) or the SNPExpress database (Heinzen et al., 2008).

**Table 1:** List of SNPs that show associations with increase risk of HLA-DQB1*06:02 negative EHS in the Japanese population.

CHR	SNP	Risk	RAF		Allelic				Dominant				Recessive				*P*_min_	Nearby Gene
		Allele	Case	Control	*P*-values	OR	L95	U95	*P*-values	OR	L95	U95	*P*-values	OR	L95	U95		
2	rs16826005	G	0.472	0.319	3.97E-06	1.89	1.43	2.50	7.51E-03	1.74	1.16	2.62	1.02E-07	3.52	2.17	5.70	1.02E-07	*NCKAP5*
2	rs12471007^*^ ^#^	C	0.456	0.312	1.33E-05	1.86	1.41	2.46	1.86E-02	1.72	1.15	2.56	1.09E-07	4.11	2.55	6.60	1.09E-07	*NCKAP5*
15	rs11854769^*^	T	0.316	0.177	6.69E-07	2.27	1.64	3.14	7.15E-06	2.43	1.64	3.59	3.61E-04	4.07	1.78	9.31	6.69E-07	*SPRED1*
15	rs2174009	C	0.335	0.193	9.34E-07	2.24	1.62	3.10	1.02E-05	2.40	1.62	3.56	2.87E-04	3.90	1.78	8.56	9.34E-07	*SPRED1*
15	rs16966389	G	0.804	0.642	7.52E-07	2.28	1.63	3.20	6.56E-07	2.73	1.82	4.10	1.07E-02	2.91	1.24	6.86	6.56E-07	*SPRED1*
2	rs359268	C	0.584	0.431	1.16E-05	1.89	1.42	2.51	1.47E-02	1.79	1.12	2.88	1.00E-06	2.81	1.84	4.30	1.00E-06	*BCL11A*
15	rs2134333	A	0.332	0.194	1.99E-06	2.19	1.58	3.02	2.05E-05	2.32	1.57	3.44	3.52E-04	3.83	1.75	8.40	1.99E-06	*SPRED1*
5	rs7725217	C	0.352	0.223	1.96E-05	2.00	1.46	2.74	1.08E-06	2.66	1.78	3.98	4.08E-01	1.41	0.62	3.19	1.08E-06	*TAS2R1*
9	rs10988217^*^	G	0.320	0.233	4.05E-03	1.52	1.13	2.04	2.55E-01	1.25	0.85	1.85	3.43E-06	3.85	2.11	7.04	3.43E-06	*PPP2R4/CRAT*
2	rs2043234	T	0.359	0.224	8.22E-06	1.95	1.45	2.64	1.15E-03	1.91	1.29	2.83	4.46E-06	4.05	2.14	7.66	4.46E-06	*NCKAP5*

**Notes.**

RAF, risk allele frequency; OR, odd ratio; L95, U95, lower and upper confidence limits; *P*_min_, minimum *P*-value among three genetic models; NA: not applicable.

Recessive model is calculated under risk allele homozygotes versus (heterozygous and non-risk homozygotes).

Dominant model is calculated under (risk allele homozygotes and heterozygotes) versus non-risk homozygotes.

* SNP count was reconfirmed by TaqMan platform.

# SNP count adjusted for TaqMan platform.

Peta odd ratio was used to calculate odd ratio for any contingency column with counting of 0.

**Figure 1:**
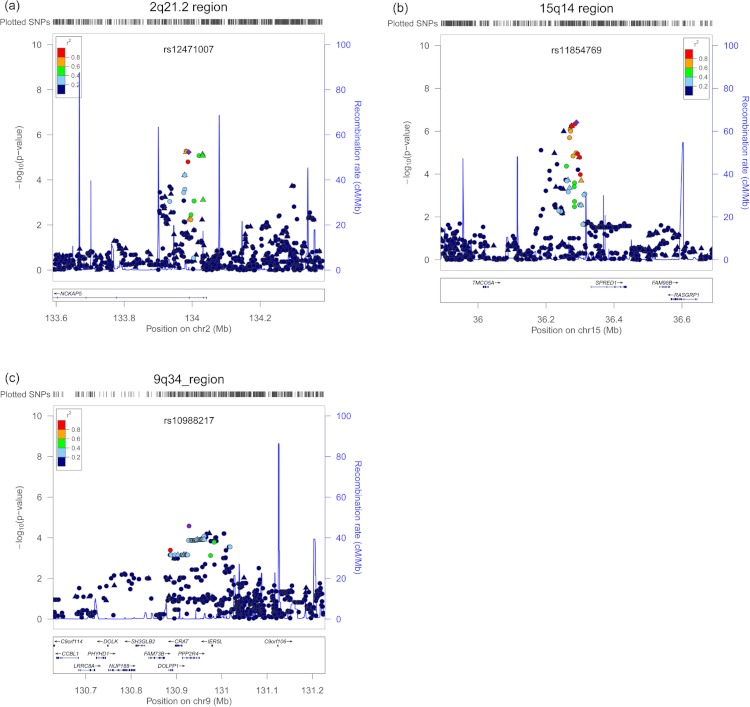
Regional association plots for three HLA-DQB1*06:02 negative EHS risk loci. (A) The region 2q21.2 Cochran–Armitage trend test *P*-value, the SNP rs16826003 is located in an intron of *NCKAP5* gene (B) The region 15q14 Cochran–Armitage trend test, SNP rs11854769 is located 42 kb upstream of the nearest gene, *SPRED1* (C) The 9q34 region based on P-minimum, rs10988217 is located in an intron of *CRAT* gene. Each of the top markers is indicated by purple diamonds. SNPs that were genotyped using Affymetrix 6.0 are marked by triangles. Imputed SNPs are plotted as circles. The color intensity represents the extent of the LD with the marker SNP, red (*r*^2^ ≥ 0.8), orange (0.6 ≤ 0.8), green (0.4 ≤ 0.6), light blue (0.2 ≤ 0.4), and dark blue (*r*^2^ ≤ 0.2). Light blue in the background indicates local recombination rate.

The SNP rs11854769 had the second lowest *P*-value by regional classification (6.69E-07, per-allele OR of 2.27 with 95% CI of 1.64–3.14) in this analysis. This SNP, rs11854769, resided within a 10 kb LD block on chromosome 15q14 (Table 1) and was located 42 kb upstream of *SPRED1* (*sprouty-related, EVH1 domain containing 1*). Imputation analysis of this region did not reveal any additional SNPs that were more significantly associated with HLA-DQB1*06:02 negative EHS (Fig. 1). An eQTL analysis showed that rs11854769 did not affect the expression level of *SPRED1* in either the GeneVar database or the SNPExpress database (Fig. S4A).

The SNP rs10988217 (*P*-value of 3.43E-06, per-allele OR of 1.52 with 95% CI of 1.13–2.04), which was located within a 63 kb LD block on chromosome 9q34.11, was also of interest (Table 1). The associated SNP is located in the intronic region of *PPP2R4* (protein phosphatase 2A activator, regulatory subunit 4) and *CRAT* (carnitine O-acetyltransferase).The eQTL analysis revealed that rs10988217 affected the expression of *CRAT* transcripts in the SNPExpress database (Fig. S4B), but not *PPP2R4* transcripts (Fig. S4C). Similar findings were observed in the GeneVar database; rs10988217 was associated with *CRAT* expression levels (*P* < 0.05) in three cell types (fibroblast, lymphoblastoid cell line and T-cell) (Fig. S3B), but not with any changes in *PPP2R4* expression (Fig. S3C).

A comparative study of Caucasian patients with HLA-DQB1*06:02 negative hypersomnia revealed that rs10988217, the SNP in the *PPP2R4*-*CRAT* region, was significantly associated with HLA-DQB1*06:02 negative hypersomnia in this population (*P*-value of 2.51E-02, per-allele OR of 1.25 with 95% CI of 1.03–1.52) (Table 2); these finds were similar to those from the GWAS of Japanese patients with HLA-DQB1*06:02 negative EHS (*P*-value of 3.43E-06, per-allele OR of 1.52 with 95% CI of 1.13–2.04) (Table 1). To investigate possible contributions of this SNP to other forms of hypersomnia, we tested the association of rs10988217 with narcolepsy with cataplexy in Japanese and Caucasian patients (Hallmayer et al., 2009). Significant associations were observed in Japanese narcolepsy (*P*-value of 2.20E-02, per-allele OR of 1.22 with 95% CI of 1.03–1.45) and Caucasian narcolepsy (*P*-value of 2.82E-02, per-allele OR of 1.13 with 95% CI of 1.01–1.27) (Table 3). Other SNPs that showed associations with an increased risk of EHS in the Japanese population were also genotyped in the samples from Caucasian patients with HLA-DQB1*06:02 negative hypersomnia, but no associations were found (Table 2).

**Table 2:** List of SNPs of interest for HLA-DQB1*06:02 negative hypersomnias samples in Caucasian population.

SNP(A/B)	Risk allele	RAF		HWE *P*-values		Chi-square 2-tailed *P*-values			OR	L95	U95	Nearest gene
		Control	Case	Control	Case	Allelic	Dominant	Recessive			
rs11854769 (C/T)	C	0.731	0.732	0.369	0.263	9.70E-01	3.00E-01	6.20E-01	1.00	0.81	1.23	*SPRED1*
rs16966290(C/A)	A	0.885	0.903	0.947	0.286	2.30E-01	7.70E-01	1.70E-01	1.20	0.88	1.63	*SPRED1*
rs12471007(C/G)	G	0.035	0.040	0.179	0.501	5.50E-01	4.40E-01	4.50E-01	1.15	0.72	1.85	*NCKAP5*
rs10988217(G/A)	G	0.597	0.648	0.581	0.456	2.51E-02	3.00E-02	1.16E-01	1.25	1.03	1.52	*CRAT*

**Notes.**

RAF: Risk allele frequency, HWE: Hardy-Weinberg Equilibrium, OR: Odds ratio, L95, U95: lower and upper limits of confidence interval at 95%.

Recessive model is calculated under risk allele homozygotes versus (heterozygous and non-risk homozygotes).

Dominant model is calculated under (risk allele homozygotes and heterozygotes) versus non-risk homozygotes.

**Table 3:** GWAS and comparative studies for rs10988217 in Japanese and Caucasian populations.

Subjects	Case count				Control count				AF (Case)		AF (Control)		Chi-Square 2-tailed *P*-value			*P*_min_	OR	L95	U95
	GG	AG	AA	Total	GG	AG	AA	Total	G	A	G	A	Allelic	Dominant	Recessive				
Japanese HLA-DQB1*06:02 negative EHS GWAS	21	38	66	125	28	206	328	562	0.320	0.680	0.234	0.770	4.05E-03	2.55E-01	3.43E-06	3.43E-06	1.52	1.13	2.04
Caucasian HLA-DQB1*06:02 negative hypersomnia	108	126	30	264	632	836	292	1760	0.648	0.352	0.597	0.403	2.51E-02	3.00E-02	1.16E-01	2.51E-02	1.25	1.03	1.52
Japanese narcolepsy	37	161	211	409	92	590	878	1560	0.287	0.713	0.250	0.750	2.22E-02	8.93E-02	2.20E-02	2.20E-02	1.22	1.03	1.45
Caucasian narcolepsy	439	503	151	1093	519	625	235	1379	0.632	0.368	0.600	0.400	3.89E-02	2.82E-02	2.00E-01	2.82E-02	1.13	1.01	1.27

**Notes.**

AF: allele frequency; OR: Odds Ratio; L95,U95: lower and upper limits of confidence interval at 95%; *P*_min_: minimum *P*-value among three genetic models.

Recessive model is calculated under risk allele homozygotes versus (heterozygous and non-risk homozygotes).

Dominant model is calculated under (risk allele homozygotes and heterozygotes) versus non-risk homozygotes.

## Discussions

This study represents the first GWAS designed to identify common genetic variants that are associated with EHS in a Japanese population; 125 individuals with EHS and 562 healthy controls participated in this study. We identified novel candidate regions associated with an increased risk of EHS in this Japanese population.

Several SNPs located in an intron of *NCKAP5* gene showed associations with an increased risk of EHS in this study. *NCKAP5* variants are reportedly strongly associated genes with bipolar disorder (Smith et al., 2009), attention deficit hyperactivity disorders (Lasky-Su et al., 2008), and multiple sclerosis (Baranzini et al., 2009). Further meta-analysis of combination of schizophrenia and bipolar disorder confirmed the association between *NCKAP5* variants and both schizophrenia and bipolar disorder (Wang, Liu & Aragam, 2010). Currently, the function of *NCKAP5* is unknown.

*SPRED1* was the gene closest to the SNP with the second highest *P*-value by regional based; *SPRED1* is a member of the Sprouty family of proteins and is known to be phosphorylated by a tyrosine kinase in response to several growth factors (Cabrita & Christofori, 2008). Proteins in the *SPRED1* family act as negative regulators of RAS-RAF interactions and of the mitogen-activated protein kinase (MAPK) signaling pathway (Brems et al., 2007). RAS/MAPK signaling has been implicated in the mediation of reversible circadian outputs (Williams et al., 2001) and sleep/wake condition mechanism (Evans, 2003) of the brain. Some narcolepsy without cataplexy patients have been reported to exhibit down-regulation of lumbar cerebrospinal fluid hypocretin-1 level (Bourgin, Zeitzer & Mignot, 2008) and hypothalamic peptides hypocretin/orexin has been reported to contribute to the intrusion of REM sleep behaviors into wakefulness by coordinating the activity of RAS through hypocretin/orexin neuron during waking stage (Burlet, Tyler & Leonard, 2002). Since *SPRED1* is an important component in the RAS pathway, *SPRED1* might play a crucial role in regulating REM sleep behaviors. In addition, germline mutations in genes involved in the RAS pathway lead to neuro-cardio-facial-cutaneous (NCFC) syndromes (ex: neurofibromatosis 1 (NF1, OMIM 162200) (Brems et al., 2007), Noonan syndrome (NS, OMIM 163950), LEOPARD syndrome (LS, OMIM 151100), cardio-facio-cutaneous syndrome (CFC, OMIM 115150), and Costello syndrome (CS, OMIM 218040) (Pasmant et al., 2009)). The SNP rs11854769 was identified in a recent GWAS of bipolar disorder (Ferreira et al., 2008); this finding may indicate that this *SPRED1* variant may confer a genetic predisposition for multiple neuropsychiatric diseases.

Association studies for rs10988217 in *CRAT* showed significant associations with both EHS and narcolepsy with cataplexy patients. In addition, these associations were observed not only in Japanese but also in Caucasians (Table 3). These results indicated that *CRAT* may be a susceptibility gene for different types of hypersomnias. The eQTL analysis of rs10988217 revealed that the SNP was associated with alterations in the expression of *CRAT* (Figs. S3B, S4B). Besides the *CRAT* association in our study, interestingly, a recent GWAS (Miyagawa et al., 2008) for narcolepsy with cataplexy in a Japanese population identified a significant association between a SNP adjacent to *CPT1B* (carnitine palmitoyltransferase 1B). This SNP is also associated with changes in *CPT1B* expression levels (Miyagawa et al., 2008). Furthermore, a subsequent association study demonstrated an association between *CPT1B* and EHS (Miyagawa et al., 2009). Both CRAT and CPT1B are involved in the β-oxidation of fatty acid. CRAT gene encodes the carnitine acetyltransferase protein, which is a key enzyme in the β-oxidation pathway in mitochondria, peroxisomes, and the endoplasmic reticulum. CRAT is responsible for catalyzing the reversible transfer of acyl compartments groups from an acyl-CoA thioester to carnitine, and this enzyme regulates the ratio of acylCoA/CoA in the subcellular compartments (Fig. S5). CPT1B is the rate-controlling enzyme of long-chain fatty acid β-oxidation in the mitochondria of muscle tissue. CPT1B catalyzes the transport of long-chain fatty acyl-CoAs from the cytoplasm into the mitochondria through the carnitine shuttle (Fig. S5). Deficiency of short-chain acyl-coenzyme A dehydrogenase in a mouse model resulted in slowing of theta frequency during REM sleep (Tafti et al., 2003). Additionally, acetyl-L-carnitine (ALCAR) is a potential treatment for neurological diseases such as Parkinson’s disease (Beal, 2003) and Alzheimer’s disease (Montgomery, Thal & Amrein, 2003); it is also known to restore β-oxidation of fatty acids in the mitochondria and rescued the slow theta frequency in REM sleep of mice lacking short-chain acyl-coenzyme A dehydrogenase (Tafti et al., 2003). Besides, our group has recently reported a clinical trial of oral L-carnitine on narcolepsy with cataplexy and the results suggested that oral L-carnitine can be a promising treatment for narcolepsy with cataplexy (Miyagawa et al., 2013; Miyagawa et al., 2011). On the basis of these reports, the results in our study indicated that the pathophysiology of hypersomnias is associated with metabolic alterations (Miyagawa et al., 2013; Miyagawa et al., 2011).

For rs10988217 in *CRAT*, the best *p*-values in Japanese EHS and narcolepsy with cataplexy patients were from the recessive model (Table 3). The recessive model was not significant in Caucasian hypersomnia and narcolepsy with cataplexy patients, but the allelic model showed a significant association (Table 3). This might be due to a difference between the populations. In addition, the risk allele (G) for rs10988217 was minor in Japanese but major in Caucasians (Table 3). As another possibility, rs10988217 is not the primary SNP of *CRAT* region. LD of the primary SNP and rs10988217 might be different between Japanese and Caucasian, contributing to the different significant model. Therefore, a further replication study and re-sequencing should be required to overcome the limitations.

## Conclusion

In summary, we report associations of *NCKAP5*, *SPRED1*, and *CRAT* variants with HLA-DQB1*06:02 negative EHS as novel candidate loci that have not been reported in other GWAS. In addition, our results showed that *CRAT* might act as a susceptibility gene for a variety of hypersomnia disorders. Further additional replication is warranted for confirmation of this study.

## Supplemental Information

10.7717/peerj.66/supp-1 Figure S1 Q–Q plot of GWAS data from the patients with HLA-DQB1*06:02 negative EHS in the Japanese population. Q–Q plot was plotted based on Conchran–Armitage trend *P*-values after standard quality control (genomic inflation factor λ = 1.008).

10.7717/peerj.66/supp-2 Figure S2 Manhattan plot of the GWAS data from the Japanese population. Manhattan plot was plotted based on *P*-value calculated using the Cochran–Armitage trend test.

10.7717/peerj.66/supp-3 Figure S3 eQTL analyses were performed based on data from the Sanger Institute GENEVAR project^14^, this expression data is based on three cell types (fibroblast, lymphoblastoid cell line, and T-cell) from 75 unrelated individuals of Western European ancestry. (A) The plot displays the relationship between *SPRED1* gene expression and rs11854769. (B) The plot displays the relationship between *CRAT* gene expression and rs10988217. (C) The plot displays the relationship between *PPP2R4* gene expression and rs10988217.

10.7717/peerj.66/supp-4 Figure S4 eQTL association analyses were performed based on transcript expression data from the SNPExpress Database; these data were derived from brain samples from 93 individuals of European ancestry (left) and Peripheral Blood Mononuclear Cell (PMBC) sample. (A) The plot displays the relationship between *SPRED1* gene expression and rs11854769. (B) The plot displays the relationship between *CRAT* gene expression and rs10988217. (C) The plot displays the relationship between *PPP2R4* gene expression and rs10988217.

10.7717/peerj.66/supp-5 Figure S5 Fatty acid synthesis, beta-oxidation and carnitine shuttle pathways in relation to *CRAT* and *CPT1B*.

## References

[2001] American Academy of Sleep Medicine, European Sleep Research Society, Japanese Society of Sleep Research, Latin American Sleep Society (2001). The international classification of sleep disorders, revised: diagnostic and coding manual.

[ref-2] American Academy of Sleep Medicine (2005). The international classification of sleep disorders: diagnostic and coding manual.

[ref-3] Baranzini SE, Wang J, Gibson RA, Galwey N, Naegelin Y, Barkhof F, Radue EW, Lindberg RL, Uitdehaag BM, Johnson MR, Angelakopoulou A, Hall L, Richardson JC, Prinjha RK, Gass A, Geurts JJ, Kragt J, Sombekke M, Vrenken H, Qualley P, Lincoln RR, Gomez R, Caillier SJ, George MF, Mousavi H, Guerrero R, Okuda DT, Cree BA, Green AJ, Waubant E, Goodin DS, Pelletier D, Matthews PM, Hauser SL, Kappos L, Polman CH, Oksenberg JR (2009). Genome-wide association analysis of susceptibility and clinical phenotype in multiple sclerosis. Human Molecular Genetics.

[ref-4] Barrett JC, Fry B, Maller J, Daly MJ (2005). Haploview: analysis and visualization of LD and haplotype maps. Bioinformatics.

[ref-5] Beal MF (2003). Bioenergetic approaches for neuroprotection in Parkinson’s disease. Annals of Neurology.

[ref-6] Bourgin P, Zeitzer JM, Mignot E (2008). CSF hypocretin-1 assessment in sleep and neurological disorders. The Lancet Neurology.

[ref-7] Brems H, Chmara M, Sahbatou M, Denayer E, Taniguchi K, Kato R, Somers R, Messiaen L, De Schepper S, Fryns JP, Cools J, Marynen P, Thomas G, Yoshimura A, Legius E (2007). Germline loss-of-function mutations in SPRED1 cause a neurofibromatosis 1-like phenotype. Nature Genetics.

[ref-8] Burlet S, Tyler CJ, Leonard CS (2002). Direct and indirect excitation of laterodorsal tegmental neurons by Hypocretin/Orexin peptides: implications for wakefulness and narcolepsy. The Journal of Neuroscience.

[ref-9] Cabrita MA, Christofori G (2008). Sprouty proteins, masterminds of receptor tyrosine kinase signaling. Angiogenesis.

[ref-10] International Hapmap Consortium (2005). A haplotype map of the human genome. Nature.

[ref-11] Dimas AS, Deutsch S, Stranger BE, Montgomery SB, Borel C, Attar-Cohen H, Ingle C, Beazley C, Gutierrez Arcelus M, Sekowska M, Gagnebin M, Nisbett J, Deloukas P, Dermitzakis ET, Antonarakis SE (2009). Common regulatory variation impacts gene expression in a cell type-dependent manner. Science.

[ref-12] Evans BM (2003). Sleep, consciousness and the spontaneous and evoked electrical activity of the brain. Is there a cortical integrating mechanism?. Neurophysiologie Clinique.

[ref-13] Ferreira MA, O’Donovan MC, Meng YA, Jones IR, Ruderfer DM, Jones L, Fan J, Kirov G, Perlis RH, Green EK, Smoller JW, Grozeva D, Stone J, Nikolov I, Chambert K, Hamshere ML, Nimgaonkar VL, Moskvina V, Thase ME, Caesar S, Sachs GS, Franklin J, Gordon-Smith K, Ardlie KG, Gabriel SB, Fraser C, Blumenstiel B, Defelice M, Breen G, Gill M, Morris DW, Elkin A, Muir WJ, McGhee KA, Williamson R, MacIntyre DJ, MacLean AW, St Clair D, Robinson M, Van Beck M, Pereira AC, Kandaswamy R, McQuillin A, Collier DA, Bass NJ, Young AH, Lawrence J, Ferrier IN, Anjorin A, Farmer A, Curtis D, Scolnick EM, McGuffin P, Daly MJ, Corvin AP, Holmans PA, Blackwood DH, Gurling HM, Owen MJ, Purcell SM, Sklar P, Craddock N, Consortium WTCC (2008). Collaborative genome-wide association analysis supports a role for ANK3 and CACNA1C in bipolar disorder. Nature Genetics.

[ref-14] Hallmayer J, Faraco J, Lin L, Hesselson S, Winkelmann J, Kawashima M, Mayer G, Plazzi G, Nevsimalova S, Bourgin P, Hong SC, Hong SS, Honda Y, Honda M, Högl B, Longstreth WT, Montplaisir J, Kemlink D, Einen M, Chen J, Musone SL, Akana M, Miyagawa T, Duan J, Desautels A, Erhardt C, Hesla PE, Poli F, Frauscher B, Jeong JH, Lee SP, Ton TG, Kvale M, Kolesar L, Dobrovolná M, Nepom GT, Salomon D, Wichmann HE, Rouleau GA, Gieger C, Levinson DF, Gejman PV, Meitinger T, Young T, Peppard P, Tokunaga K, Kwok PY, Risch N, Mignot E (2009). Narcolepsy is strongly associated with the T-cell receptor alpha locus. Nature Genetics.

[ref-15] Heinzen EL, Ge D, Cronin KD, Maia JM, Shianna KV, Gabriel WN, Welsh-Bohmer KA, Hulette CM, Denny TN, Goldstein DB (2008). Tissue-specific genetic control of splicing: implications for the study of complex traits. PLoS Biology.

[ref-16] Honda Y, Juji T, Matsuki K, Naohara T, Satake M, Inoko H, Someya T, Harada S, Doi Y (1986). HLA-DR2 and Dw2 in narcolepsy and in other disorders of excessive somnolence without cataplexy. Sleep.

[ref-17] Juji T, Satake M, Honda Y, Doi Y (1984). HLA antigens in Japanese patients with narcolepsy. All the patients were DR2 positive. Tissue Antigens.

[ref-18] Komada Y, Inoue Y, Mukai J, Shirakawa S, Takahashi K, Honda Y (2005). Difference in the characteristics of subjective and objective sleepiness between narcolepsy and essential hypersomnia. Psychiatry and Clinical Neurosciences.

[ref-19] Lasky-Su J, Neale BM, Franke B, Anney RJ, Zhou K, Maller JB, Vasquez AA, Chen W, Asherson P, Buitelaar J, Banaschewski T, Ebstein R, Gill M, Miranda A, Mulas F, Oades RD, Roeyers H, Rothenberger A, Sergeant J, Sonuga-Barke E, Steinhausen HC, Taylor E, Daly M, Laird N, Lange C, Faraone SV (2008). Genome-wide association scan of quantitative traits for attention deficit hyperactivity disorder identifies novel associations and confirms candidate gene associations. American Journal of Medical Genetics Part B: Neuropsychiatric Genetics.

[ref-20] Li Y, Willer CJ, Ding J, Scheet P, Abecasis GR (2010). MaCH: using sequence and genotype data to estimate haplotypes and unobserved genotypes. Genetic Epidemiology.

[ref-21] Marcadet A, Gebuhrer L, Betuel H, Seignalet J, Freidel AC, Confavreux C, Billiard M, Dausset J, Cohen D (1985). DNA polymorphism related to HLA-DR2 Dw2 in patients with narcolepsy. Immunogenetics.

[ref-22] Mignot E, Lin L, Rogers W, Honda Y, Qiu X, Lin X, Okun M, Hohjoh H, Miki T, Hsu S, Leffell M, Grumet F, Fernandez-Vina M, Honda M, Risch N (2001). Complex HLA-DR and -DQ interactions confer risk of narcolepsy-cataplexy in three ethnic groups. The American Journal of Human Genetics.

[ref-23] Miyagawa T, Honda M, Kawashima M, Shimada M, Tanaka S, Honda Y, Tokunaga K (2009). Polymorphism located between CPT1B and CHKB, and HLA-DRB1*1501-DQB1*0602 haplotype confer susceptibility to CNS hypersomnias (essential hypersomnia). PLoS ONE.

[ref-24] Miyagawa T, Honda M, Kawashima M, Shimada M, Tanaka S, Honda Y, Tokunaga K (2010). Polymorphism located in TCRA locus confers susceptibility to essential hypersomnia with HLA-DRB1*1501-DQB1*0602 haplotype. Journal of Human Genetics.

[ref-25] Miyagawa T, Kawamura H, Obuchi M, Ikesaki A, Ozaki A, Tokunaga K, Inoue Y, Honda M (2013). Effects of oral L-carnitine administration in narcolepsy patients: a randomized, double-blind, cross-over and placebo-controlled trial. PLoS ONE.

[ref-26] Miyagawa T, Kawashima M, Nishida N, Ohashi J, Kimura R, Fujimoto A, Shimada M, Morishita S, Shigeta T, Lin L, Hong SC, Faraco J, Shin YK, Jeong JH, Okazaki Y, Tsuji S, Honda M, Honda Y, Mignot E, Tokunaga K (2008). Variant between CPT1B and CHKB associated with susceptibility to narcolepsy. Nature Genetics.

[ref-27] Miyagawa T, Miyadera H, Tanaka S, Kawashima M, Shimada M, Honda Y, Tokunaga K, Honda M (2011). Abnormally low serum acylcarnitine levels in narcolepsy patients. Sleep.

[ref-28] Montgomery SA, Thal LJ, Amrein R (2003). Meta-analysis of double blind randomized controlled clinical trials of acetyl-L-carnitine versus placebo in the treatment of mild cognitive impairment and mild Alzheimer’s disease. International Clinical Psychopharmacology.

[ref-29] Mukai J, Inoue Y, Honda Y, Takahashi Y, Ishii A, Saitoh K, Nanba K (2003). Clinical characteristics of essential hypersomnia syndrome. Sleep and Biological Rhythms.

[ref-30] Pasmant E, Sabbagh A, Hanna N, Masliah-Planchon J, Jolly E, Goussard P, Ballerini P, Cartault F, Barbarot S, Landman-Parker J, Soufir N, Parfait B, Vidaud M, Wolkenstein P, Vidaud D, France RN (2009). SPRED1 germline mutations caused a neurofibromatosis type 1 overlapping phenotype. Journal of Medical Genetics.

[ref-31] Pruim RJ, Welch RP, Sanna S, Teslovich TM, Chines PS, Gliedt TP, Boehnke M, Abecasis GR, Willer CJ (2010). LocusZoom: regional visualization of genome-wide association scan results. Bioinformatics.

[ref-32] Purcell S, Neale B, Todd-Brown K, Thomas L, Ferreira MA, Bender D, Maller J, Sklar P, de Bakker PI, Daly MJ, Sham PC (2007). PLINK: a tool set for whole-genome association and population-based linkage analyses. The American Journal of Human Genetics.

[ref-33] Roth B (1976). Narcolepsy and hypersomnia: review and classification of 642 personally observed cases. Schweiz Arch Neurol Neurochir Psychiatr.

[ref-34] Smith EN, Bloss CS, Badner JA, Barrett T, Belmonte PL, Berrettini W, Byerley W, Coryell W, Craig D, Edenberg HJ, Eskin E, Foroud T, Gershon E, Greenwood TA, Hipolito M, Koller DL, Lawson WB, Liu C, Lohoff F, McInnis MG, McMahon FJ, Mirel DB, Murray SS, Nievergelt C, Nurnberger J, Nwulia EA, Paschall J, Potash JB, Rice J, Schulze TG, Scheftner W, Panganiban C, Zaitlen N, Zandi PP, Zöllner S, Schork NJ, Kelsoe JR (2009). Genome-wide association study of bipolar disorder in European American and African American individuals. Molecular Psychiatry.

[ref-35] Tafti M, Petit B, Chollet D, Neidhart E, de Bilbao F, Kiss JZ, Wood PA, Franken P (2003). Deficiency in short-chain fatty acid beta-oxidation affects theta oscillations during sleep. Nature Genetics.

[ref-36] Wang KS, Liu XF, Aragam N (2010). A genome-wide meta-analysis identifies novel loci associated with schizophrenia and bipolar disorder. Schizophrenia Research.

[ref-37] Williams JA, Su HS, Bernards A, Field J, Sehgal A (2001). A circadian output in Drosophila mediated by neurofibromatosis-1 and Ras/MAPK. Science.

[ref-38] Yang TP, Beazley C, Montgomery SB, Dimas AS, Gutierrez-Arcelus M, Stranger BE, Deloukas P, Dermitzakis ET (2010). Genevar: a database and Java application for the analysis and visualization of SNP-gene associations in eQTL studies. Bioinformatics.

