# Supplementary figures and images for: Genome-wide association study of HLA-DQB1*06:02 negative essential hypersomnia
Source: PeerJ. 2013 Apr 16;1:e66. doi: 10.7717/peerj.66 (PMC3642778; doi:10.7717/peerj.66)

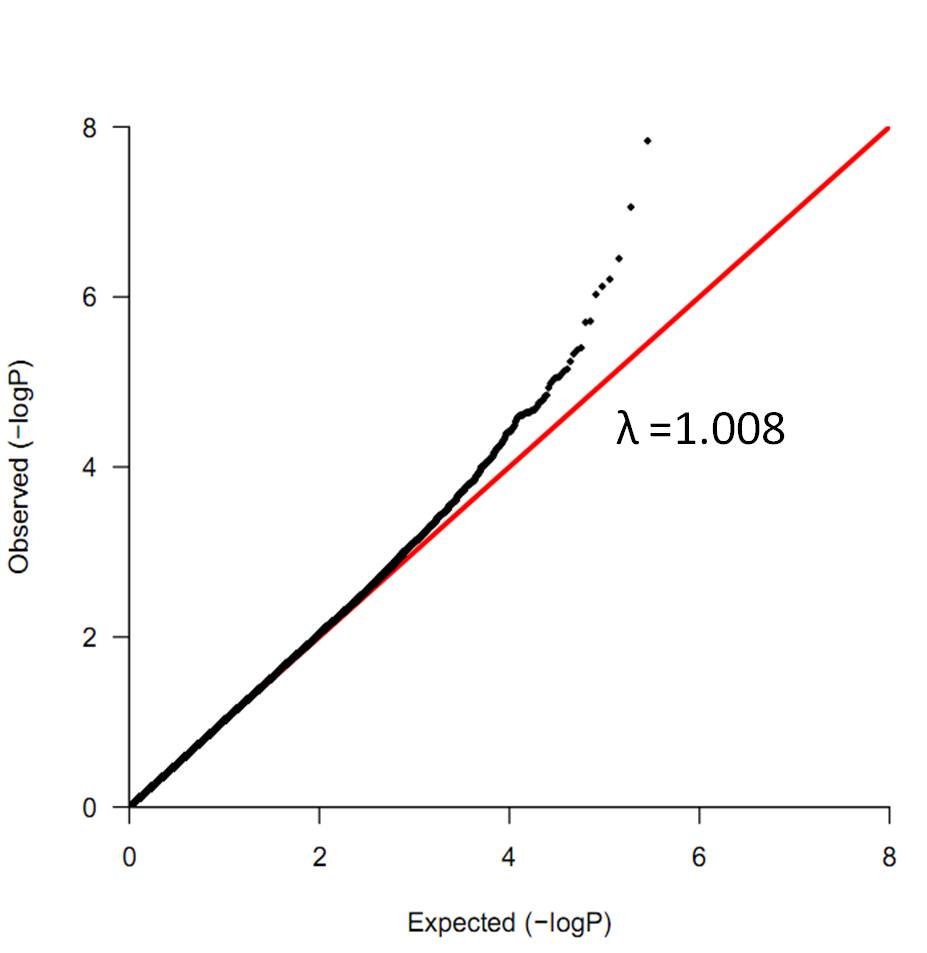

Supplement: Figure S1 [file peerj-01-66-s001.jpg]

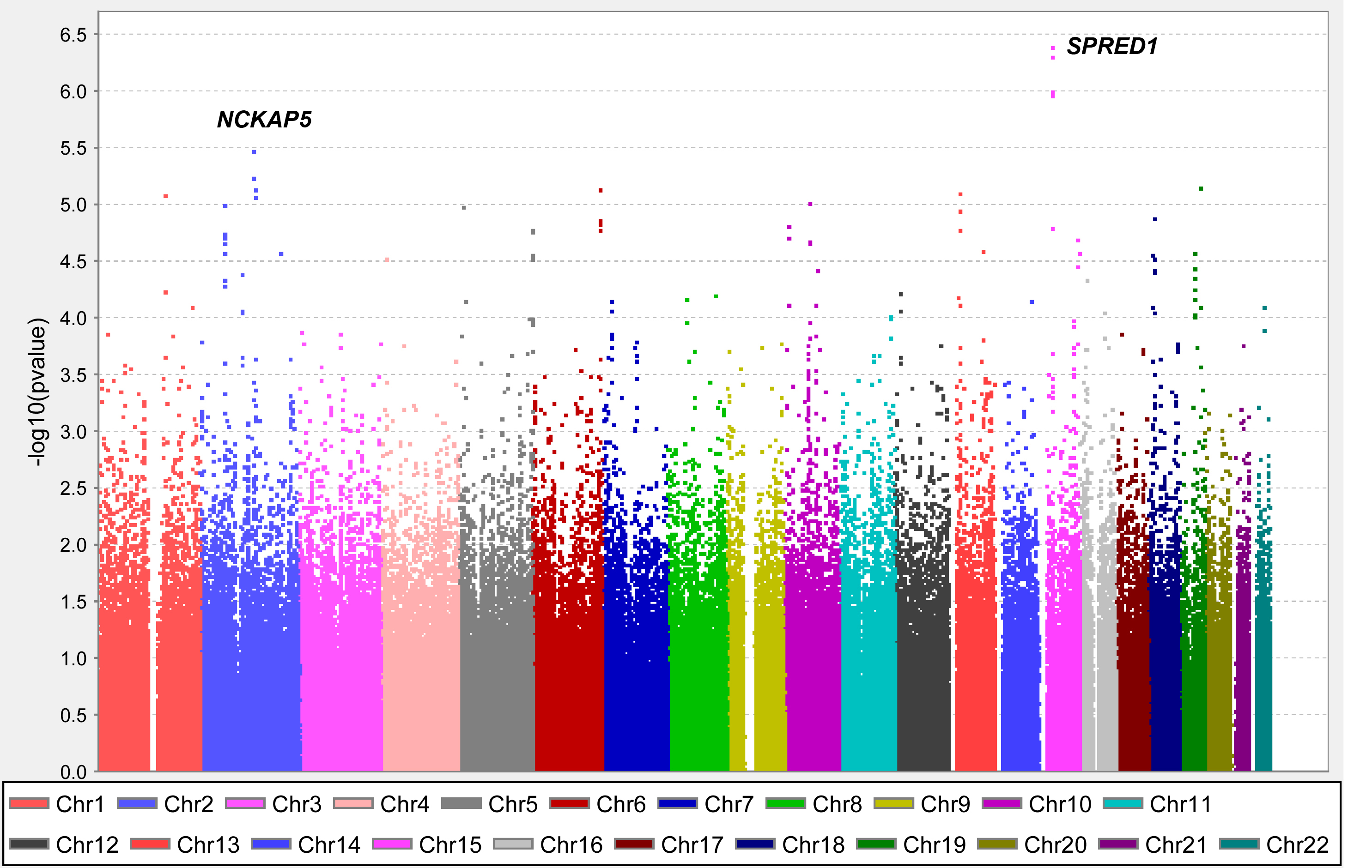

Supplement: Figure S2 — Manhattan plot was plotted based on P-value calculated using the Cochran–Armitage trend test. [file peerj-01-66-s002.jpg]

4 (a)

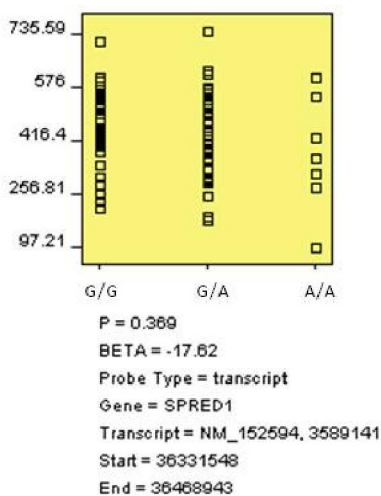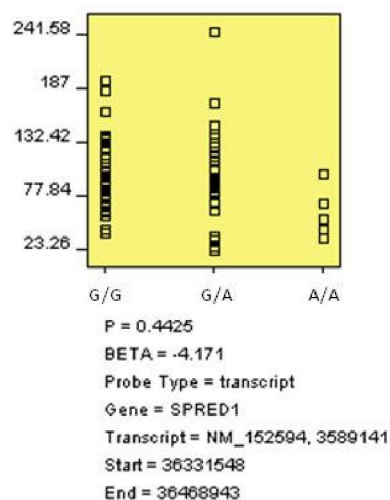

4 (b)

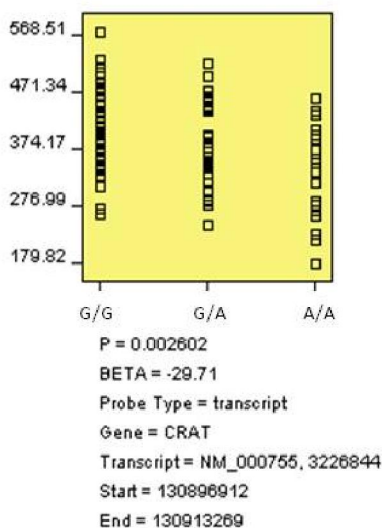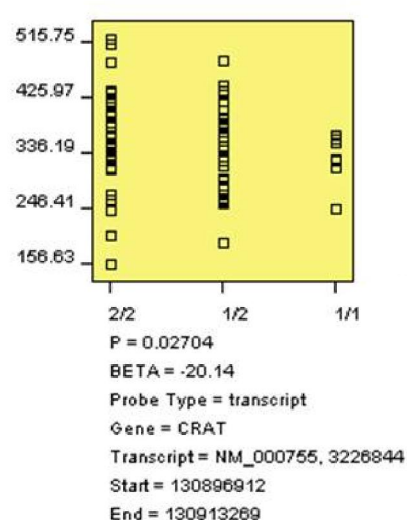

4 (c)

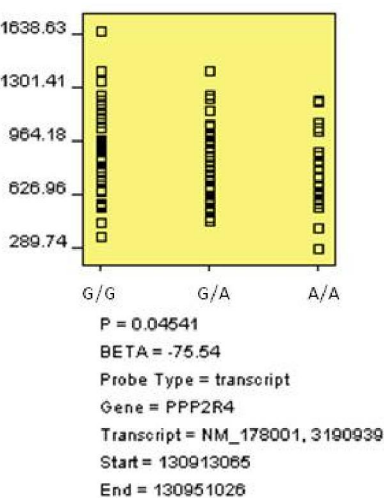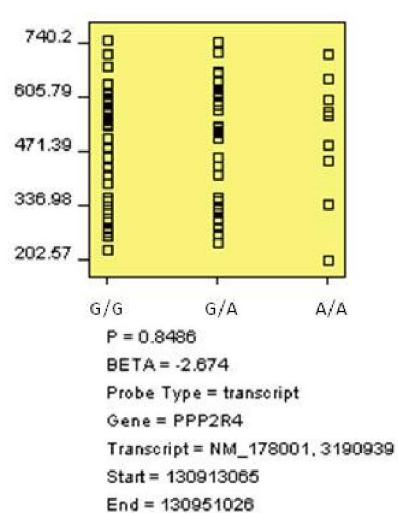

Supplement: Figure S4 — eQTL association analyses were performed based on transcript expression data from the SNPExpress Database; these data were derived from brain samples from 93 individuals of European ancestry (left) and Peripheral Blood Mononuclear Cell (PMBC) sample. (A) The plot displays the relationship between SPRED1 gene expression and rs11854769. (B) The plot displays the relationship between CRAT gene expression and rs10988217. (C) The plot displays the relationship between PPP2R4 gene expression and rs10988217. [file peerj-01-66-s004.pdf]
