# Supplementary material for: Genome-wide association study of HLA-DQB1*06:02 negative essential hypersomnia
Source: PeerJ. 2013 Apr 16;1:e66. doi: 10.7717/peerj.66 (PMC3642778; doi:10.7717/peerj.66)

3 (a) Expression level of *SPRED1* gene against rs11854769 SNP marker

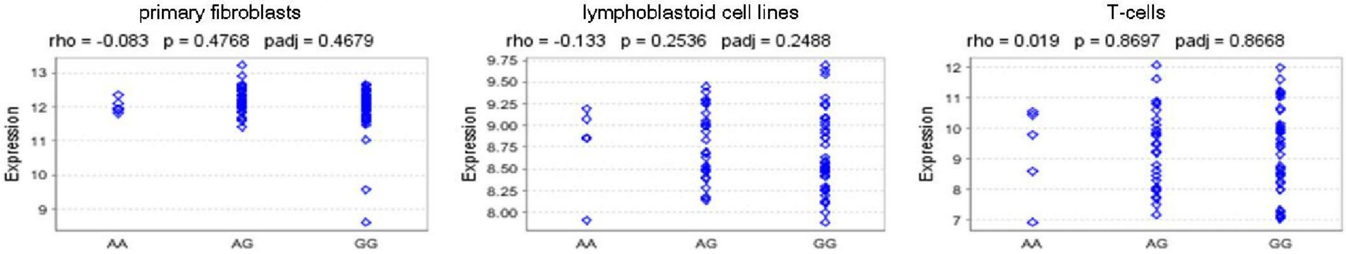

3 (b) Expression level of *CRAT* gene against rs10988217 SNP marker

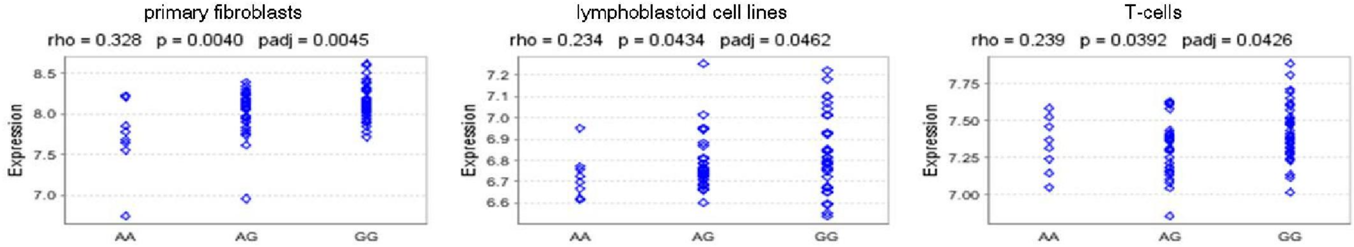

3 (c) Expression level of *PPP2R4* gene against rs10988217 SNP marker

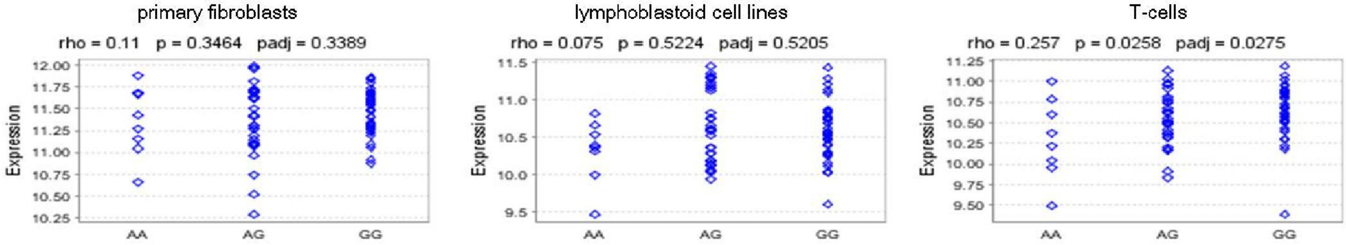

Supplement: Figure S3 — eQTL analyses were performed based on data from the Sanger Institute GENEVAR project14, this expression data is based on three cell types (fibroblast, lymphoblastoid cell line, and T-cell) from 75 unrelated individuals of Western European ancestry. (A) The plot displays the relationship between SPRED1 gene expression and rs11854769. (B) The plot displays the relationship between CRAT gene expression and rs10988217. (C) The plot displays the relationship between PPP2R4 gene expression and rs10988217. [file peerj-01-66-s003.pdf]
