# Supplementary material for: Genome-wide association study of HLA-DQB1*06:02 negative essential hypersomnia
Source: PeerJ. 2013 Apr 16;1:e66. doi: 10.7717/peerj.66 (PMC3642778; doi:10.7717/peerj.66)
Supplement: Figure S5 [file peerj-01-66-s005.pdf]

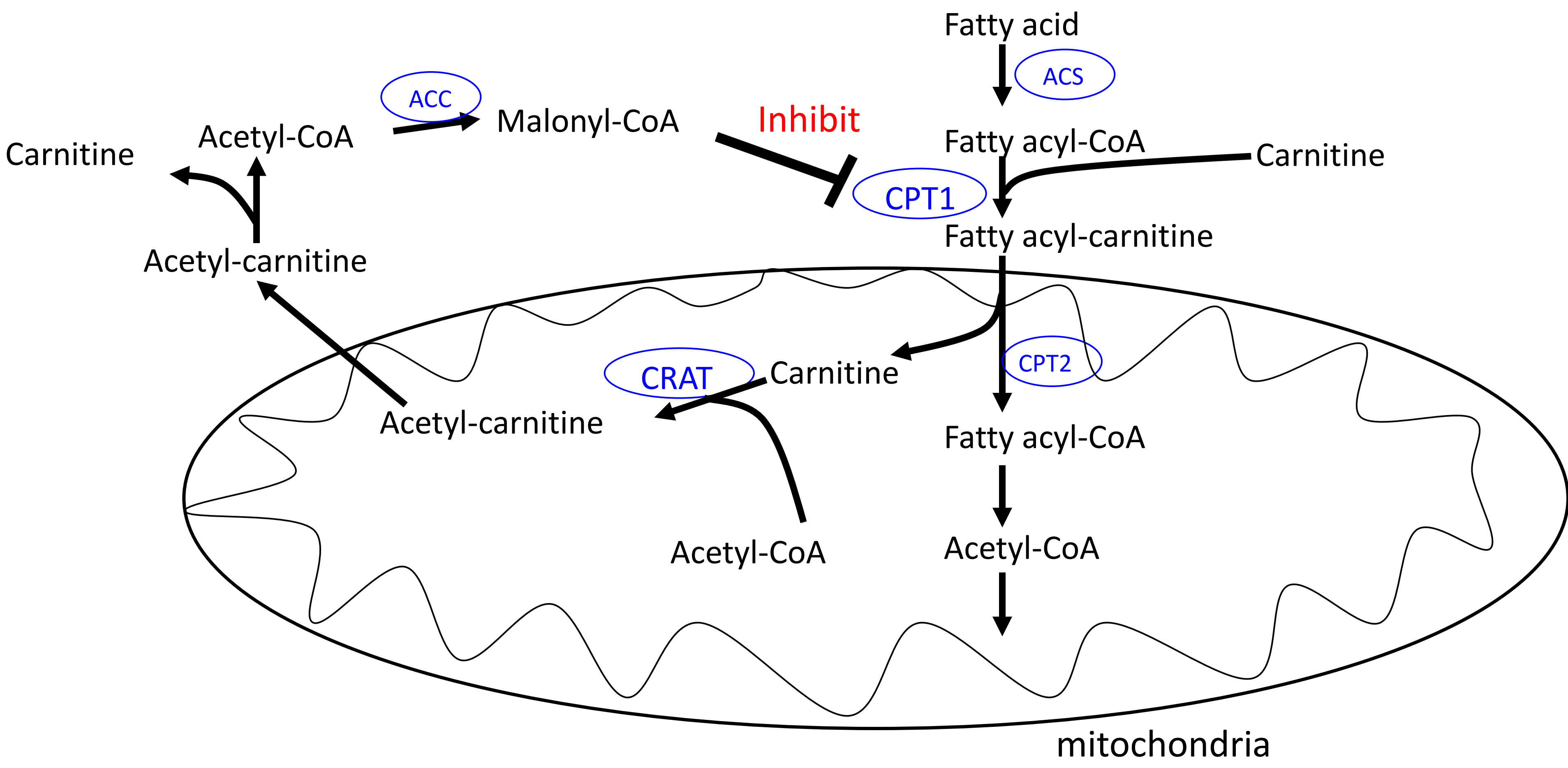

ACC: acetyl-CoA carboxylase, ACS: acyl-CoA synthase,
CPT1: carnitine palmitoyltransferase I , CPT2: carnitine palmitoyltransferase 2,
CRAT: carnitine acetyltransferase
